# Pathological Impact on the Phyllosphere Microbiota of *Artemisia argyi* by Haze

**DOI:** 10.4014/jmb.2009.09024

**Published:** 2021-02-12

**Authors:** Yu-Zhu Zhang, De-Yu Jiang, Chi Zhang, Kun Yang, Huai-Fu Wang, Xiu-Wen Xia, Wei-Jun Ding

**Affiliations:** 1 Department of Fundamental Medicine, Chengdu University of Traditional Chinese Medicine, 1166 Liutai Avenue, Chengdu 611137, P.R. China; 2 College of Clinical Medicine, Chengdu University of Traditional Chinese Medicine, Chengdu 610072, P.R. China; 3 Zigong Hospital of Traditional Chinese Medicine, 59 Ma Chongkou Street, Zigong 643010, P.R. China; 4 Health Preservation and Rehabilitation College, Chengdu University of Traditional Chinese Medicine, Chengdu 611137, P.R. China

**Keywords:** Haze, particulate matter (PM2.5), *Artemisia argyi*, phyllosphere microbiota, metagenomics

## Abstract

The pathological impact of haze upon the phyllosphere microbiota awaits investigation. A moderate degree of haze environment and a clean control were selected in Chengdu, China. *Artemisia argyi*, a ubiquitously distributed and extensively applied Chinese herb, was also chosen for experiment. Total genome DNA was extracted from leaf samples, and for metagenome sequencing, an Illumina HiSeq 2500 platform was applied. The results showed that the gene numbers of phyllosphere microbiota derived from haze leaves were lower than those of the clean control. The phyllosphere microbiota derived from both haze and clean groups shared the same top ten phyla; the abundances of Proteobacteria, Actinomycetes and Anorthococcuso of the haze group were substantially increased, while Ascomycetes and Basidiomycetes decreased. At the genus level, the abundances of *Nocardia*, *Paracoccus*, *Marmoricola* and *Knoelia* from haze leaves were markedly increased, while the yeasts were statistically decreased. KEGG retrieval demonstrated that the functional genes were most annotated to metabolism. An interesting find of this work is that the phyllosphere microbiota responsible for the synthesis of primary and secondary metabolites in *A. argyi* were significantly increased under a haze environment. Relatively enriched genes annotated by eggNOG belong to replication, recombination and repair, and genes classified into the glycoside hydrolase and glycosyltransferase enzymes were significantly increased. In summary, we found that both structure and function of phyllosphere microbiota are globally impacted by haze, while primary and secondary metabolites responsible for haze tolerance were considerably increased. These results suggest an adaptive strategy of plants for tolerating and confronting haze damage.

## Introduction

The pathological impact of haze on plant leaves has not been fully explored. Air pollutants, represented by haze, have a substantial impact on human beings, animals and plants. Conceptually, haze is usually referred to as an atmospheric phenomenon caused by a mixture of diverse pollution sources, especially particulate matter with a diameter of 2.5 μm or less (PM2.5). Increasing research has indicated that along with the increase of PM2.5, the prevalence of asthma, chronic obstructive pulmonary disease, lung cancer and cardiovascular diseases has grown rapidly [1]. Beyond its impact on human beings, haze can also inhibit plant growth and result in wide-ranging damage. From Mississippi to Brazil, air pollution has prevented and debilitated the growth of vegetable crops and rainforests [2]. Exposure to a high concentration of aerial SO_2_ can impair the antioxidant mechanisms of spruce trees [3]. DNA damage to *Euonymus japonicus* collected roadside was found to be significantly higher than that in its non-roadside counterparts and was positively correlated with the severity of haze [4].

For a long time, studies on phyllosphere microbiota have lagged far behind those focusing on rhizosphere microbiota, partially due to the fact that the latter might play a more important role in crop production, based on earlier studies. However, facilitated by ready sample collection, easy observation and short research periods, phyllosphere microbiota have gradually become a subject of interest for probing the damage mechanisms of haze upon plants [5, 6]. Under continuing stress from haze, the dynamic characteristics of the phyllosphere microbiota can be overlooked. In a haze environment, different sensitivity/resistance in various plants might lead to distinctive patterns in the phyllosphere microbiota, and consequently influence the relationship with the host, the plant leaves. Increasing studies have demonstrated that haze can injure both the structure and function of phyllosphere microbiota. The abundances of *Staphylococcus*, *Sphenoaminolomonas* and *Polaromonas* within the phyllosphere microbiota of roadside-growing *Nerium oleander* and *Osmanthus fragrans* were significantly increased compared to those living in a clean area [7]. Duarte *et al*. [8] observed a drop off of four times in the fungal diversity index in phyllosphere microbiota after a plant was placed in polluted condition. The diversity of phyllosphere microbiota may be linked to adaptability to the haze environment. Those sensitive to a haze environment will gradually decrease or disappear, while the abundance of the haze-adapted species will increase slowly, thus slightly changing the structure and function of phyllosphere microbiota.

A majority of Chinese herbs fall within the botanical kingdom, sharing a similar interactive pattern between the leaf tissue and phyllosphere microbiota [9]. Intensive cooperation and competition among medicinal plants and their symbiotic phyllosphere microbiota might be a main evolutionary cause behind the synthesis and accumulation of secondary metabolisms, the active components of Chinese herbs [10]. However, few studies have been conducted on the pathological damage to Chinese herbs caused by haze. *A. argyi*, a ubiquitously distributed herb extensively used in Chinese medicine, was chosen as a representative herb to explore the pathological mechanisms confronting haze. We therefore applied a high-throughput sequencing platform in this study to explore the structural and functional differences of the phyllosphere microbiota of *A. argyi* derived from a haze environment and a clean area as control. This work may provide novel evidence for the pathological damages caused by haze to Chinese herbs and alert the public to the possibly of lower quality *A. argyi* collected from polluted environments.

Dimension reduction analysis can reduce information noise and prominent differences. Representative methods, such as principal component analysis (PCA), principal coordinate analysis (PCoA) and dimensionless multidimensional calibration (NMDS), have been extensively used in the data processing areas of metagenomics, metabonomics, and proteomics. PCA, for instance, reduces the data dimensions through variance decomposition, and reflects them on a two-dimensional coordinate graph [11-13].

## Materials and Methods

### Grouping

**Grouping criteria**. A moderate degree of haze environment is defined as having an air quality index (AQI) between 201-250 [14], while the representative haze environment refers to places where annually, days of middle-degree haze are detected 100 ± 10 times. On the other hand, the clean area applied in this study was defined as having an annual average AQI of < 50. Three years of AQI data were supplied by the Environment Protection Bureau (http://www.cdepb.gov.cn/cdepbws/web/gov/airquality.aspx), Chengdu, China. The daily AQIs are officially collected every ten square kilometers in Chengdu. These data have been released daily since July 01, 2010.

**Sampling places**. Based on the above criteria, two places were recruited for sampling collection. The area of No. 37 Shier Qiao Road, within the 1^ST^ Ring Road of downtown Chengdu, China, was selected as middle-degree haze environment. This location is known for its high energy consumption and heavy traffic. Contrarily, No. 108, Shi-Su Road, Chengdu, China, which was chosen as our clean control, is located within a wetland park with various species of wildlife and is far away from factories or roads.

### Sample Collection and Genome DNA Isolation

On December 15, 2017, leaf samples of mature *A. argyi* were collected. Sterile masks and gloves were worn by our team members during the whole examination period. Forceps, scissors, beakers and other articles for sample collection were packaged separately before sterilization. The leaf samples were picked and put into a high-temperature sterilized jar. Yellow, senescent, or insect egg-contaminated leaves were excluded. Based on our preliminary test, 100 g and 250 g of leaf samples were collected in haze and clean areas, respectively. These samples were identified as cultivated *A. argyi* by Dr. Liu Wei, College of Pharmacy, Chengdu University of Traditional Chinese Medicine.

In order to isolate and collect the microbes on the leaf surface, cotton swabs soaked in 0.8% normal saline solution (NS) were used. Both sides of each collected leaf was gently wiped by cotton swabs which were immediately placed into 100 ml of 0.8% NS, followed by centrifugation at 500 rpm for 30 min to fully dilute the microbes adhered to the swab, followed by centrifugation at 3,000 rpm for 15 min to collect the microbe pad. Three repeated samples representing either haze group (*i.e.*, EG1, EG2, EG3) or clean group (*i.e.*, CG1, CG2, CG3) were obtained. All obtained samples of total DNA were stored at -80°C for follow-up analysis.

Total genome DNA from each sample was extracted by hexadecyl trimethyl ammonium bromide and Sodium Dodecyl Sulfate (CTAB/SDS) method. The concentration and integrity of the extracted DNA were monitored by 1% agarose gel electrophoresis, and adjusted to 1 ng/μl using sterile water. Then, these samples of genome DNA were sent to a commercial company for metagenomic analysis.

### Metagenome Sequencing

Our team used the same research platform to complete the phyllosphere microbiota study for this topic [15]. For metagenomic sequencing, DNA concentration was measured using a dsDNA Assay Kit in 2.0 Flurometer (Life Technologies, USA). A total amount of 1 μg DNA per sample was used as input material for the DNA sample preparations. Sequencing libraries were generated using an Ultra DNA Library Prep Kit for Illumina (NEB, USA) following the manufacturer’s instructions. Briefly, the DNA sample was fragmented by sonication to about 350 bp, then DNA fragments were end-polished, A-tailed and ligated with the full-length adaptor for Illumina sequencing with further PCR amplification. As a last step, PCR products were purified (AMPure XP System) and libraries were analyzed for size distribution by an Agilent2100 Bioanalyzer and quantified using real-time PCR. The clustering of the index-coded samples was performed on a cBot Cluster Generation System according to the manufacturer’s instructions. After cluster generation, the library preparations were sequenced on an Illumina HiSeq 2500 platform. The scaftigs information obtained after all the sequencing was assembled for quality control for further analysis.

### Bioinformatics Analysis

Raw data of the shotgun metagenomic sequencing were continuously split, assembled, filtrated and the chimera was removed [11, 17, 18]. Then, using the UPARSE software, we analyzed the sequences data on Operational Taxonomic Units (OTUs) production and sequences with 97% similarity were assigned to the same OTUs. Representative sequences for each OTU were screened for further annotation. The Ribosomal Database Project 3 Classifier was used to conduct further analysis based on OTU clustering and species annotation (Version 2.2). Statistical tests on the taxonomic differences between samples were calculated by Welch’s t-test combined with Welch’s inverted method for calculating confidence intervals (nominal coverage of 95%). The metagenomic data were compared with three representative databases: Kyoto Encyclopedia of Genes and Genomes (KEGG), evolutionary genealogy of genes: Non-supervised Orthologous Groups (eggNOG), and Carbohydrate-Active Enzymes Database (CAZy). These databases integrate genomic, chemical and functional information to annotate the phyllosphere microbiota.

### Statistical Analysis

SPSS software (V16.0; SPSS Inc., USA) was used for statistical analyses. All data were expressed as the mean ± SD. Data were analyzed with one-way analysis of variance (ANOVA). Differences between the groups were evaluated using Student–Newman–Keuls. Data were considered to be statistically significant with each value of *p < 0.05 or **p < 0.01.

The raw data of 16S rRNA sequencing has been presented and is available at SRA (SRR11285575, SRR11285574, SRR11285573, SRR11285572, SRR11285571, SRR11285576). https://www.ncbi.nlm.nih.gov/bioproject/PRJNA611026

## Results

### Gene Prediction and Sample Correlation Analysis

The quality of the original data was evaluated with the Illumina HiSeq 2500. The results showed that the percentages of valid data over the original data were ranged from 98.906% to 99.418% (Table 1), indicating the extremely high quality of these sequencing data. The core-pan analysis was executed as follows: scaftigs were obtained by assembling valid data derived from scaffolds, other reads were assorted packing with the same parameters as scaffold (Table 2). These assembling or packing fragments of more than 500 bp were statistically analyzed, and relevant genes were then predicted (Table 3) [12].

The results of the core-pan analysis showed there were 652,138 and 675,468 genes or OTUs observed in the phyllosphere microbiota of haze and clean group, respectively. The shared gene number of both groups was 513,460. The gene number of phyllosphere microbiota derived from haze leaves was lower than that of clean control, suggesting a decreased diversity in the haze environment. Relevant coefficient heatmap analysis demonstrated that the bacteria abundance within the same group was highly similar, compared with the different expressed genes (Fig. S1).

### Species Annotation Displays Statistical Differences of Haze Group

**Fig. 1:**
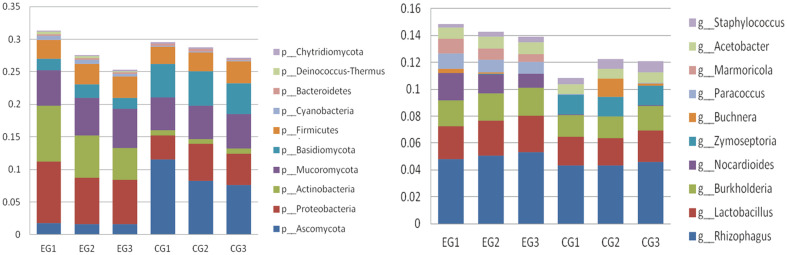
Histogram of the top ten abundance specimens. Left: top ten phyla. Right: top ten genera. EG: haze group. CG: clean group.

Based on the relative abundances of diverse taxonomic levels, the top ten specimens were respectively selected, while the remains were included as “Others”. Fig. 1 shows the annotation results of phylum (left) and genus level (right).

Both phyllosphere microbiota from haze and clean group shared the same top ten phyla, *i.e.*, Actinobacteria, Proteobacteria, Ascomycota, Bacidiomycota, Mucoromycota, Cyanobacteria, Deinococcus Thermus, Firmicutes, Bacteroidetes, and Chytridiomycota (Table 4). Compared with clean group, the abundances of Proteobacteria, Actinomycetes and *Anorthococcus thermozoophya* of the haze group were significantly increased, while Ascomycetes and Basidiomycetes were significantly decreased. Interestingly, the fungi abundance from haze environment was markedly decreased than that of clean group, while the abundance of bacteria statistically increased.

At the genus level, the abundances of *Nocardia*, *Paracoccus*, *Marmoricola* and *Knoelia* in the phyllosphere microbiota of haze group were significantly increased, while the yeast were statistically decreased (Table 5). Abundances of other genera displayed changes somewhat, yet did not reach statistical difference (Fig. 1).

**Fig. 2:**
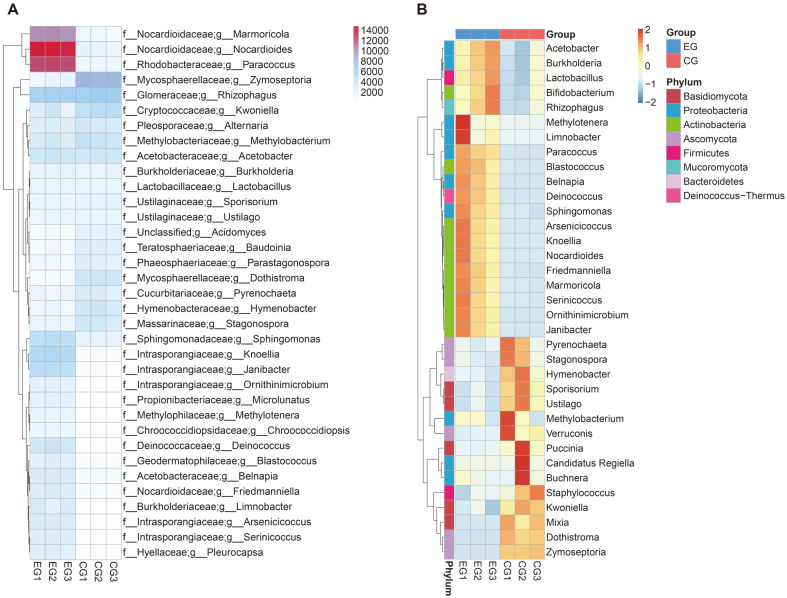
Clustering heatmap between the haze and clean group. Left: the phylum level of relative abundance. Right: the genus level of relative abundance. The numbers in the middle of heatmaps were the Z value normalized in each row. EG: haze group. CG: clean group.

Depending on the above abundance information, the top 35 specimens were selected. The clustering heatmap and clustering map were drawn in order to directly show the differences between the haze and the clean groups of other phylum and genus levels (Fig. 2). These heatmaps displayed significant differences between the haze group and clean group.

**Fig. 3:**
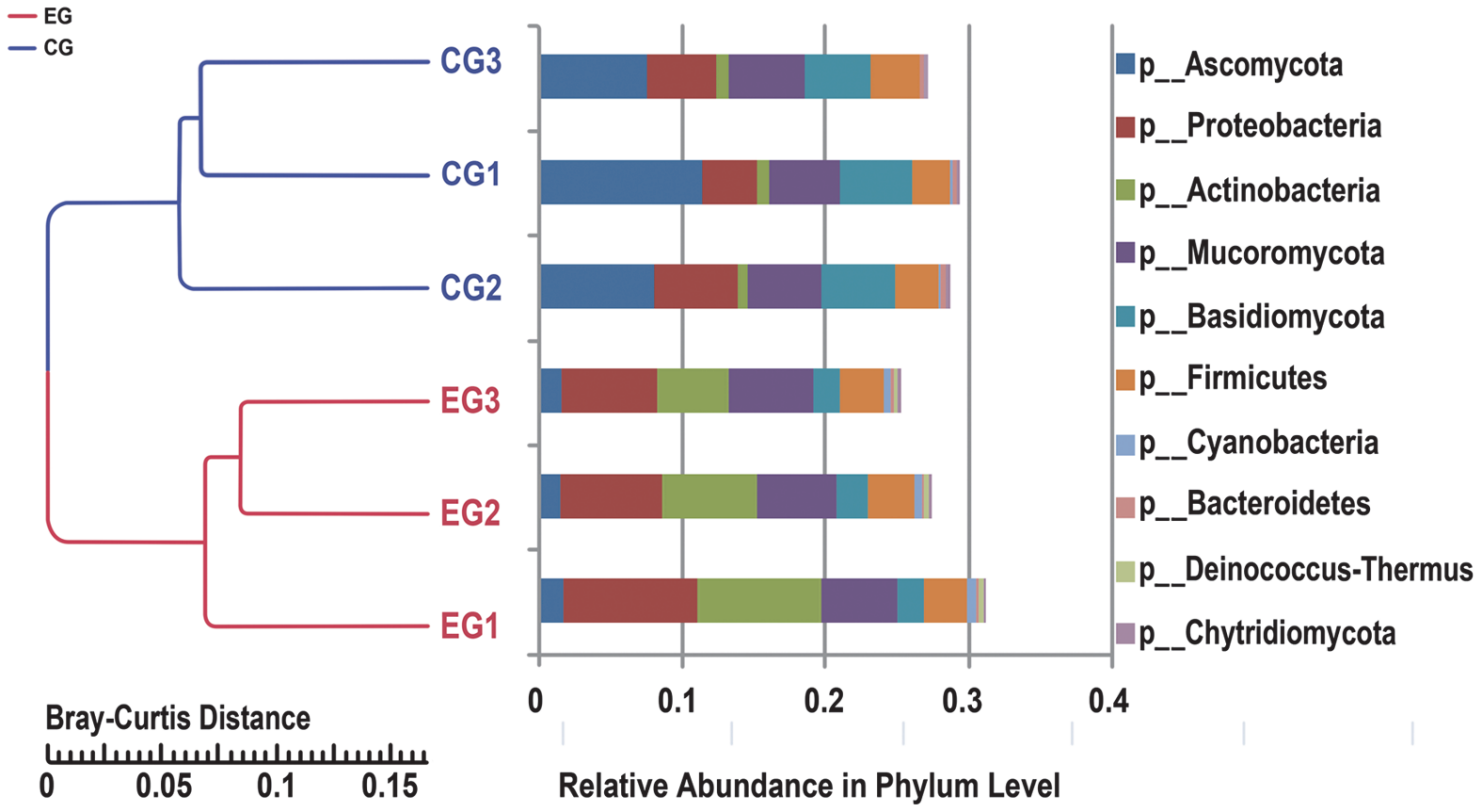
Clustering tree based on Bray-Curtis distance. The left side is the Bray-Curtis distance clustering tree structure; the right side is the relative abundance distribution map of each sample at the phylum level, and the right-most legend represents different species categories at the phylum level. EG: haze group. CG: clean group.

To investigate the similarity of both groups, the Bray-Curtis distance clustering tree was constructed according to the information on abundance (Fig. 3). At the phylum level, rather far distance between the haze group and the clean group indicated significant differences between the haze and clean groups (*p* = 0.001).

### Function Annotation of the Phyllosphere Microbiota

**Fig. 4:**
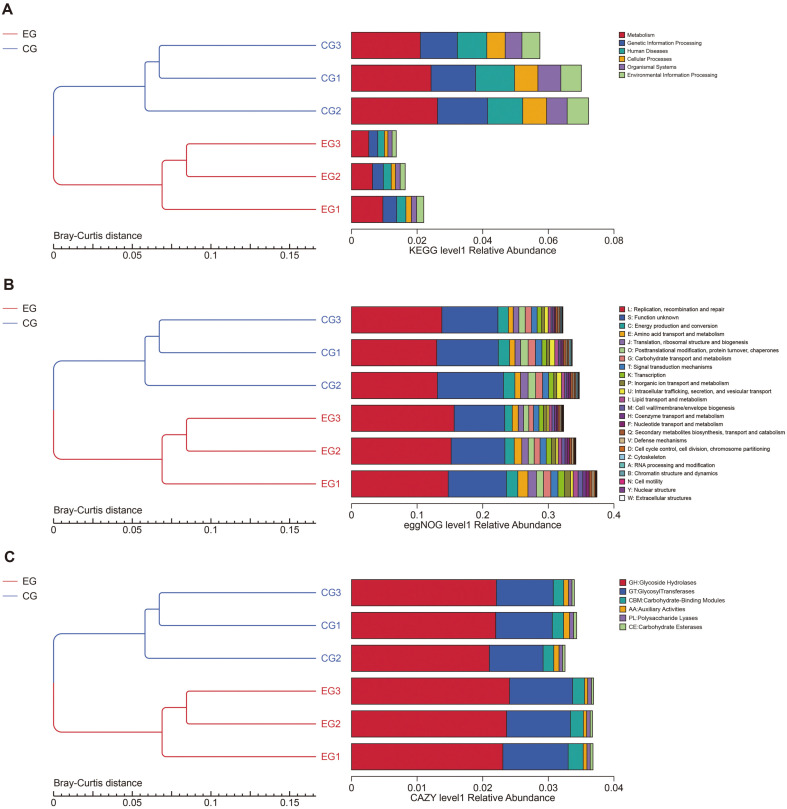
Clustering tree based on Bray-Curtis distance. The left side is the Bray-Curtis distance clustering tree structure; the right side is the distribution of functional relative abundance of each sample at the first level of each database. The legend on the far right represents the specific functions under the first classification. Upper: Results of KEGG analysis. Middle: Results of eggNOG analysis. Lower: Results of CAZy analysis. EG: haze group. CG: clean group.

To investigate constructive similarities of recruited samples, the Bray-Curtis distance cluster tree was constructed based on three classical databases, KEGG, eggNOG and CAZy. Results of these database analyses showed significant functional differences between haze and clean group (Fig. 4).

Results of KEGG retrieval demonstrated that the functional genes were most annotated to metabolisms, accounting for about 2% in the first level of relative abundance (Fig. 4A). Compared with the clean group, the number of functional genes annotated to metabolism, genetic information processing, human diseases, cell processes, biological systems, and environmental information processing was significantly reduced in haze samples. One interesting find of this work is that the phyllosphere microbiota genes responsible for the synthesis of primary and secondary metabolites in *A. argyi* were significantly increased under haze environment (*p* = 0.001). Results of KEGG analysis demonstrated diverse metabolic pathways involving xenobiotics biodegradation and metabolism, metabolism of terpenoids and polyketides, glycan biosynthesis and metabolism, carbohydrate metabolism, metabolism of cofactors and vitamins, and biosynthesis of other secondary metabolites.

Relative enriched genes annotated by the eggNOG database belonged to replication, recombination and repair, accounting for about 12% of all annotated genes (Fig. 4B). Compared with clean group, genes for replication, recombination and repair annotated from the haze group were markedly increased. Annotated functional genes responsible for RNA processing and modification, chromatin structure and dynamics, intracellular transport, secretion and vesicle transport, nuclear structure and cytoskeleton, were significantly induced in the phyllosphere microbiota derived from haze leaves of *A. argyi*.

Forty-four percent of functional genes annotated by the CAZy database belonged to glycoside hydrolases (Fig. 4C). Genes classified into glycoside hydrolase and glycosyltransferase enzymes in the haze group were markedly increased, while genes for carbohydrate binding modules and auxiliary activities were statistically decreased.

**Fig. 5:**
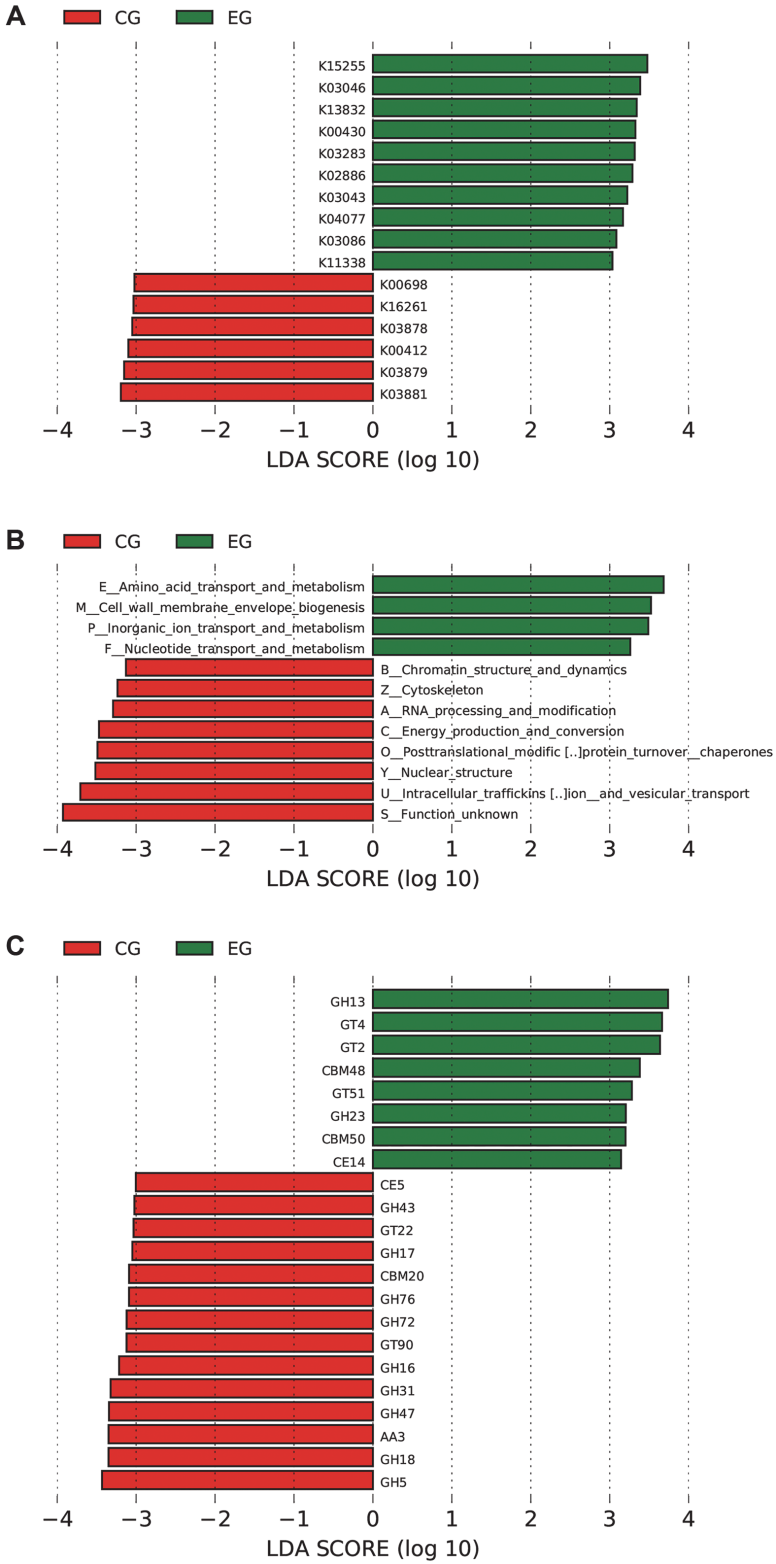
Distribution of LDA values derived from KEGG, eggNOG and CAZy analysis. The length of the histogram represents the impact of differential function (that is, LDA Score). EG: The haze group. CG: The clean group. Upper: Results of KEGG analysis. Middle: Results of eggNOG analysis. Lower: Results of CAZy analysis. LDA: (linear discriminant analysis).

To find the functional biomarkers responsible for haze impact, the Wilcoxon rank sum test was used to detect differentially expressed genes between the haze group and clean control. The dimension reduction was achieved by LDA (linear discriminant analysis) method. The distribution of LDA values of functional genes was illustrated in Fig. 5. Based on KEGG analysis, the functional genes K15255, K03046, K13832, K03283, K02886, K03043, K04077, K03086 and K11338 were significantly expressed in the haze group, corresponding to genes *PIF1, rpoC, aroDE, HSPA1S, MRPL2, rpoB, HSPD1, rpoD, RUVBL2*. In the clean group, the K03881, K03879, K00412, K03878, K16261, and K00698 functional genes were significantly expressed, corresponding to genes *ND4, ND2, CYTB, ND1, YAT, CHS1*. Results of eggNOG data mining showed that the functions of amino acid transport and metabolism, cell wall membrane envelope biogenesis, inorganic ion transport and metabolism, nucleotide transport and metabolism were significantly expressed in the haze group. After retrieval from the CAZy database, *GH13, GT4, GT2, CBM48, GT51, GH23, CBM50, CE14* and other functional genes showed significant overexpression in the haze group, compared with those of clean control, reflecting the haze-induced activation process of sugar hydrolysis and synthesis.

## Discussion

The diversity of phyllosphere microbiota in *A. argyi* was decreased under haze environment, while microbes with resistance to haze impact were increased. Compared with the clean group, the fungi abundance derived from haze group was decreased while the bacteria diversity was significantly increased. This may be related to nutrient competition between different microbes and various degrees of sensitivity or resistance to haze pollution. Fenn *et al*.[19] found that the diversity of phyllosphere fungi of giant cedar (Western Arborvitae) could be altered confronting ozone and sulfur dioxide. Artificial aerosol can markedly reduce the diversity of bacteria, filamentous fungi, and yeast in mango leaves [20]. However, Brighigna and colleagues [21] demonstrated that air pollution could increase the diversity index of phyllosphere fungi in *Tillandsia*. Haze and other air pollutants can cause serious damage to leaf and phyllosphere microbiota symbiosis. Hence, immovable plants have to evolve special mechanisms for surviving in deleterious atmospheric conditions. Under the long-term impact of air pollutants, the abundance of sensitive species will be reduced or disappear, while those with resistance or tolerance features will survive and/or evolve. Such dynamic processes continuously change the structure of phyllosphere microbiota. Our research demonstrated that the abundances of *Nocardia*, *Paraphylococcus*, *Marmoricola*, *Knoellia* and *Sphingomonas* from the haze samples were significantly increased. Some studies found that these species exhibited characteristics relevant to anti-pollutants [7, 21-23]. The strain ATD6 isolated from farmland soil and belonging to the genus *Nocardia* could effectively degrade melamine by co-culture with strains CDB21 and CSB1 [22]. It was established that certain species of *Paracoccus* have strong ability for pollutant degradation; *Pseudomonas* species *P. solvenotivorans* can degrade acetone, *P. aminovorans* and *P. aminophilus* can utilize dimethyl formamide, and *P. thiocyanatus* can use ester thiocyanate as growth substrate [24]. The haze-degrading properties of the over-growth phyllosphere species derived from haze environment need to be further explained.

An interesting find of this work is that the genes responsible for the synthesis of primary and secondary metabolites in phyllosphere microbiota of *A. argyi* were significantly increased under haze environment. Results of KEGG analysis demonstrated various metabolic pathways, majorly involved in xenobiotics biodegradation and metabolism, metabolism of terpenoids and polyketides, glycan biosynthesis and metabolism, metabolism of cofactors and vitamins, and biosynthesis of other secondary metabolites. This suggests that when confronting haze, a major environmental challenge, the phyllosphere microbiota could promote adaptive processes, and consequently, globally promote the metabolism for producing and accumulating metabolites against haze impact. Primary and secondary metabolites of plants are usually produced and accumulated while confronting abiotic stress or pathogen infection, which could improve a plant’s survival ability. For instance, the concentrations of primary metabolites and phenolic compounds of jujube seedlings were markedly increased after exposure to acute ozone [25]. Long-term ozone exposure can promote the total phenol level of capsaicin, and consequently, reduce the antioxidant activity of capsaicin [26]. Ultraviolet radiation can alter the morphological and physiological features of certain plants, partly due to the reduction of secondary metabolites that can protect plants through the quenching effect of reactive oxygen and nitrogen [27]. Secondary metabolites of medicinal plants can accumulate under environmental stress [28]. Under the action of high temperature and heavy metal stress, locust seedlings can enhance their protection and defense activities through the accumulation of secondary metabolites [29]. Linear discriminant (LEfSe) analysis of functional differences between groups showed that most of the genes significantly expressed in the haze group played an important role in improving the environmental adaptability of plants. PIF gene is involved in the resistance and adaptability of plants to abiotic stresses such as high temperature, low temperature and drought. The *rpoC* is mainly involved in DNA repair and recombination. The *aroDE* is mainly involved in metabolic pathways, biosynthesis of secondary metabolites and biosynthesis of amino acids. HSPA1S is involved in environmental information processing. The *rpoB* is involved in gene information processing, DNA repair and recombination. HSPD1 is mainly involved in RNA degradation. Therefore, the accumulation of primary and secondary metabolites in the phyllosphere microbiota of *A. argyi* can be considered as an adaptive process against the major challenge of the haze environment.

Metagenomic results indicate the phyllosphere microbiota of *A. argyi* can tolerate the impact of haze by genetic regulation. Functional genes annotated by eggNOG displayed that, in haze group, those responsible for DNA replication, recombination and repair were significantly increased. Some of these annotated genes have been investigated for their tolerant and/or resistant properties. Under high temperature stress, genes involved in DNA replication and metabolism were up-regulated [30]. Over-expression of helicase, which mediates DNA replication, recombination and repair, can improve plant tolerance to abiotic stress [31]. After ultraviolet treatment, the expression of MSH2 and MSH6 proteins in *Arabidopsis thaliana* were significantly increased, suggesting that the dimer formed by MSH2 and MSH6 may repair the DNA damaged by UV, thus keeping the plant genome stable [32]. OsRad9.1 plays an important role in gene repair, which will significantly increase in rice under stress conditions such as drought, salt and heavy metals [33]. A growing number of studies have explored increasing kinds of tolerance and resistance mechanisms for enduring air pollution, in which the ability of gene replication, recombination and repair play important roles. Haze, as a novel abiotic environmental stressor, may share some tolerant mechanisms with UV radiation, high temperature, drought, high salt, heavy metals, and other stressors. On the other hand, as a deleterious chemical mixture of aerosol, haze likely contains special pathological mechanisms that remain for further elucidation.

The relatively small size of samples is one of the shortcomings of this work, which might not represent the complex status of haze environment. Secondly, only one season, the winter, was observed in this work. Even if the winter is the most serious period of haze impact in Chengdu, China, other seasons in the studied area can also exhibit different patterns of haze pollution. Thirdly, limited reference publications restricted the metagenomic analysis of the phyllosphere microbiota. And finally, in order to evaluate the haze impact on the medicinal compounds of *A. argyi*, the metabolite profile of the leaves and their immunological mechanisms with the phyllosphere microbiota need to be probed. Such endeavors may also benefit improving the quality and quantity of Chinese herbs.

In summary, we found that the structure and function of the phyllosphere microbiota were globally impacted by haze, wihle the primary and secondary metabolites of *A. argyi* responsible for haze tolerance were significantly increased. This suggests a novel adaptive strategy of certain Chinese herbs in tolerating damage caused by haze.

## Supplemental Materials



Supplementary data for this paper are available on-line only at http://jmb.or.kr.

## Figures and Tables

**Table 1:** Statistical results of pretreated data.

#	Insert Size (bp)	Raw Data	Clean Data	Clean_Q20	Clean_Q30	Clean_ GC (%)	Effective (%)
EG2	350	9,134.59	9,055.49	96.79	91.77	46.48	99.134
EG3	350	8,181.28	8,113.68	96.75	91.72	44.7	99.174
CG2	350	8,154.29	8,032.49	97.23	92.66	41.49	98.506
CG3	350	9,225.51	9,171.83	97.22	92.66	41.16	99.418
EG1	350	6,570.88	6,517.05	96.84	92	48.62	99.181
CG1	350	7,201.15	7,122.40	96.92	92.04	43.27	98.906

Note: #Sample name. Insert Size (BP): A 350 bp library was used. Raw Data: Data derived from the sequencing machine. Clean Data: the filtered and valid data. Clean_Q20: the percent of the clean data with a sequencing error rate less than 0.01(mass value greater than 20). Clean_Q30: the percent of the clean data with a sequencing error rate less than 0.001(mass value greater than 30). Clean_GC (%): the percentage of GC among total base pairs in clean data. Effective (%): the percentage of clean data among raw data.

**Table 2:** Assembly results of basic scaftigs.

#	Total Len.	Num.	Average Len. (bp)	N50 Len. (bp)	N90 Len. (bp)	Max Len. (bp)
EG1	137,922,383	188,565	731.43	695	526	51,710
EG2	237,882,177	319,777	743.9	722	531	30,249
EG3	212,608,709	288,685	736.47	713	530	31,611
CG1	287,019,410	294,700	973.94	925	546	46,717
CG2	334,529,617	359,310	931.03	883	542	31,809
CG3	365,892,032	387,766	943.59	892	547	96,133
NOVO_MIX	442,508,741	595,063	743.63	727	532	4,987

Note: NOVO_MIX: mixed assembly result. Total Len. (BP): the total length of assembled Scaftigs. Num.: the total number of assembled Scaftigs. Average Len.: the Average length of Scaftigs in certain sample. N50 Len.: Rank scaftigs by length when reached 50% of the total Scaftigs. N90 Len.: Rank scaftigs by length when reached 90% of the total Scaftigs. Max Len.: the longest one among the assembled scaftigs.

**Table 3:** Statistical results of predicted genes.

Sample name	ORFs NO.	Integrity: none	Integrity:end	Integrity:start	Integrity:all TotalLen.	Average Len.	GC (%)
CG1	255,845	14,854(5.81%)	63,223(24.71%)	61,251(23.94%)	116,517(45.54%) 108.43	423.82	51.12
CG2	277,438	13,379(4.82%)	71,854(25.9%)	66,326(23.91%)	125,879(45.37%) 114.28	411.93	49.43
CG3	298,881	13,204(4.42%)	74,159(24.81%)	66,638(22.3%)	144,880(48.47%) 119.18	398.76	49.44
EG1	142,124	17,527(12.33%)	40,104(28.22%)	43,527(30.63%)	40,966(28.82%) 56.17	395.19	55.08
EG2	218,877	23,326(10.66%)	62,774(28.68%)	63,462(28.99%)	69,315(31.67%) 79.94	365.21	53.35
EG3	180,784	15,950(8.82%)	52,356(28.96%)	50,576(27.98%)	61,902(34.24%) 61.9	342.39	50.08
NOVO_MIX	374,239	34,643(9.26%)	109,297(29.21%)	104,678(27.97%)	125,621(33.57%) 123.45	329.87	51.32

Note: ORFs NO.: the number of genes annotated in the Gene Catalogue. Integrity:start: the percentage of annotated genes that contain the start codon only. Integrity:end: the percentage of annotated genes that contain the end codon only. Integrity:none: the percentage of genes that have neither a start codon nor end codon. Integrity:all: the percentage of completed genes which have both start and end codons. Total length (Mbp): the Total length of genes annotated from the Gene Catalogue that represent in millions of base pairs. Average Len.: the average length of the sequenced genes within a sample. GC(%): the percent of the total GC base pairs among the predicted genes.

**Table 4:** The relative abundances between groups at phylum level.

Phylum	p__Ascomycota	p__Proteobacteria	p__Actinobacteria	p__Mucoromycota	p__Basidiomycota	p__Firmicutes	p__Cyanobacteria	p__Bacteroidetes	p__Deinococcus-Thermus	p__Chytridiomycota	Others
EG1	0.017271065	0.094873125	0.085703279	0.054231275	0.01753928	0.029043103	0.006884065	0.00194528	0.00379416	0.001696525	0.687018844
EG2	0.016079253	0.071240361	0.064773027	0.057406656	0.021341226	0.031443955	0.006167864	0.001963745	0.003157623	0.001813151	0.724613139
EG3	0.015687019	0.068232478	0.048796682	0.060114663	0.017007731	0.032611283	0.004550404	0.001786035	0.002307226	0.001917558	0.746988921
CG1	0.114919706	0.037459187	0.008040359	0.049933623	0.051265325	0.026930885	0.001347441	0.00319634	9.50E-05	0.00199966	0.704812482
CG2	0.082093931	0.056858244	0.007926375	0.050540418	0.053032603	0.029411959	0.001518389	0.003825266	9.07E-05	0.002420369	0.712281758
CG3	0.076144647	0.048320525	0.008046634	0.052469513	0.047462633	0.033136543	0.001546592	0.002240365	8.19E-05	0.001804629	0.728746018

Note: EG: The haze group. CG: The clean group.

**Table 5:** The relative abundances between groups at genus level.

Genus	g__Rhizophagus	g__Lactobacillus	g__Burkholderia	g__Nocardioides	g__Zymoseptoria	g__Buchnera	g__Paracoccus	g__Marmoricola	g__Acetobacter	g__*Staphylococcus*	Others
EG1	0.047773645	0.024626111	0.019139714	0.020306415	0.000307443	0.002861148	0.011511131	0.011209417	0.008296319	0.002681154	0.851287502
EG2	0.050727003	0.025670441	0.020373257	0.014210763	0.000348894	0.000966421	0.009486719	0.008317583	0.00883829	0.004006879	0.857053749
EG3	0.053154839	0.026923726	0.021183149	0.010036566	0.000323287	3.60E-05	0.008520743	0.005878114	0.009119424	0.003972789	0.860851347
CG1	0.04323713	0.021144431	0.016564625	0.000355859	0.014491381	0.000290914	0.000175763	0.00024123	0.007409172	0.00456106	0.891528436
CG2	0.043085061	0.020591092	0.015947226	0.000317398	0.014470868	0.013296952	0.000148351	0.000200284	0.007102371	0.007295102	0.877545294
CG3	0.046055409	0.023264163	0.018319972	0.000196547	0.014952108	0.001664235	0.00013687	0.000115106	0.007890543	0.008367227	0.87903782

Note: EG: The haze group. CG: The clean group.
