# Supplementary material for: Pathological Impact on the Phyllosphere Microbiota of Artemisia argyi by Haze
Source: J Microbiol Biotechnol. 2021 Feb 12;31(4):510–9. doi: 10.4014/jmb.2009.09024 (PMC9723278; doi:10.4014/jmb.2009.09024)
Supplement: Supplementary file 1 [file jmb-31-4-510-supple.pdf]

J. Microbiol. Biotechnol.
https://doi.org/10.4014/jmb.2009.09024

Supplementary Figure

Correlation analysis between samples:

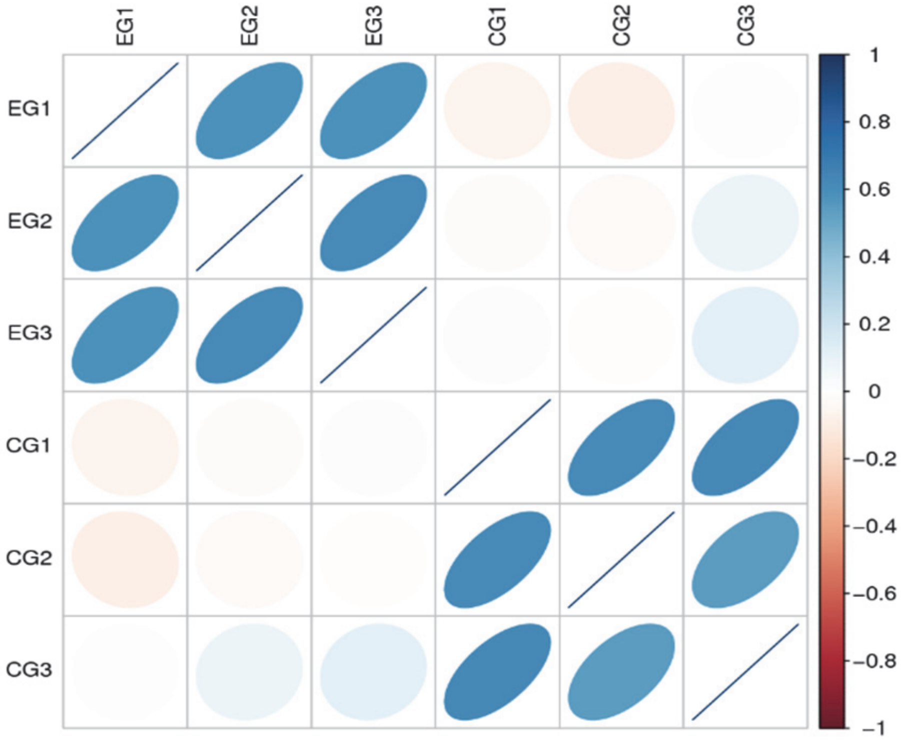

Figure S1: Heat diagram of correlation coefficient between samples. EG: The haze group. CG: The clean group.
